# Immunoglobulin A Antibodies: From Protection to Harmful Roles

**DOI:** 10.1111/imr.13424

**Published:** 2024-11-23

**Authors:** Patrick J. Gleeson, Niels O. S. Camara, Pierre Launay, Agnès Lehuen, Renato C. Monteiro

**Affiliations:** ^1^ Center for Research on Inflammation Paris Cité University Paris France; ^2^ INSERM Paris France; ^3^ CNRS Paris France; ^4^ Inflamex Laboratory of Excellence Paris France; ^5^ Nephrology Department Bichat Hospital Paris France; ^6^ Department of Immunology, Institute of Biomedical Sciences University of Sao Paulo Sao Paulo Brazil; ^7^ Cochin Institute, INSERM, CNRS Paris Cité University Paris France

**Keywords:** immunoglobulin A, inflammation, microbiota, mucosal immunity

## Abstract

Immunoglobulin A (IgA) is the most abundantly produced antibody in humans. IgA is a unique class of immunoglobulin due to its multiple molecular forms, and a defining difference between the two subclasses: IgA1 has a long hinge‐region that is heavily O‐glycosylated, whereas the IgA2 hinge‐region is shorter but resistant to bacterial proteases prevalent at mucosal sites. IgA is essential for immune homeostasis and education. Mucosal IgA plays a crucial role in maintaining the integrity of the mucosal barrier by immune exclusion of pathobionts while facilitating colonization with certain commensals; a large part of the gut microbiota is coated with IgA. In the circulation, monomeric IgA that has not been engaged by antigen plays a discrete role in dampening inflammatory responses. Protective and harmful roles of IgA have been studied over several decades, but a new understanding of the complex role of this immunoglobulin in health and disease has been provided by recent studies. Here, we discuss the physiological and pathological roles of IgA with a special focus on the gut, kidneys, and autoimmunity. We also discuss new IgA‐based therapeutic approaches.

## 1 Introduction

Mucosal surfaces represent a large part of the human body displaying a sophisticated interactive immune system with environmental factors. During evolution, it became essential for mammal development to create several layers in physical barrier structures allowing mucosal tolerance toward microbiota as well as active defensive mechanisms against external antigens [1, 2]. Among multiple actors, immunoglobulin A (IgA) emerged as a major player [3]. IgA was first described by Gugler and Heremans in the late 1950s after their observations of human milk and unusual myeloma proteins [4, 5]. IgA is the predominant antibody class present in mucosal areas, such as the gastrointestinal, respiratory, and urogenital tracts, where it plays a crucial role in immune tolerance and immune protection. Not only IgA acts in immune exclusion defense mechanisms but also in mutualistic interactions of the adaptive immune system with mucosal microbial to shape their compositions [6].

IgA is also significantly present in plasma although its role during systemic immune responses toward external antigens appears to be less important as compared to IgG or IgM antibodies [7, 8].

## 2 Molecular Basis of IgA

In humans, there are two subclasses of IgA: IgA1 and IgA2 [9, 10]. IgA2 has two allotypes, known as IgA2m (1) and IgA2m (2) [11, 12]. A third variant of IgA2, termed IgA2(n), has been described; it is likely a product of allelic recombination between the IgA2m (1) and IgA2m (2) heavy chains. The heavy chains of these immunoglobulins are encoded by IGHA1 and IGHA2 on chromosome 14. Overall, there are only 22 differences in amino acid residues between IgA1 and IgA2, mostly found in the hinge‐region. IgA1 represents about 90% of serum IgA while the other 10% is made up of IgA2. The ratio of IgA1 to IgA2 in colostrum is 65:35, in saliva 60:40, in jejunal fluid 70:30, and in the colon 35:65 [13].

Nonhuman primates such as Chimpanzees, Gorillas, and Gibbons also have 2 IgA subclasses [14]. Logamorphs, including rabbits, have up to 15 IgA heavy chain genes, although only 11 expressed forms have been characterized [15]. In mice and rats, only one IgA form exists.

### 2.1 IgA Structure

In contrast to other human Ig classes, IgA can be found in multiple molecular forms with a variable distribution in multiple body fluids. Monomeric (m) IgA dominates in blood, where polymers are only present at low percentages [7, 13, 16]. The molecular mass of mIgA is 160 kDa. Both subclasses of IgA have a pair of either κ or λ light chains, which are 25 kDa and are made up of a variable (V_L_) domain and a constant (C_L_) domain. The heavy chains are composed of a variable domain (VH) and three constant domains (Cα1, Cα2, and Cα3) going from N‐terminus to C‐terminus.

Similar to IgM, the heavy chains of IgA have an 18 amino acid extension at the C‐terminal end of Cα3, known as the tailpiece. This is essential for the ability of IgA to bind J chain and form IgA polymers (pIgA) [17, 18]. Each heavy chain is ~55 kDa in size. The V_L_ and C_L_ domains of each light chain line up with the V_H_ and Cα1 domains of each heavy chain, respectively, forming two Fab regions. One important difference between the IgA subtypes lies in the way the Fab regions are stabilized. In IgA1 this is by a disulfide bridge between the C_L_ domain and Cys133 of Cα1, while in IgA2m (2) the LC is joined to Cys 220 of Cα1. The IgA2m (1) heavy chain is unusual, as it does not form any disulfide bond with the light chain; instead, the light chains form a disulfide bond between each other, and the Fab region is stabilized by noncovalent bonds between heavy and light chains; so, under denaturing conditions, IgA2m (1) divides into light chain and heavy chain dimers [19].

The IgA in mucosal secretions is exclusively produced in a dimeric form [20]. Dimeric IgA interacts with the polymeric Ig receptor (pIgR) expressed on basolateral compartments of epithelial cells. This binding allows for selective transport of IgA through the epithelial cells [20]. Comparative structural studies of the amino‐acid sequences of IgA1 and IgA2 H chains revealed major differences in their hinge regions. The IgA2 H‐chain has a shorter hinge region due to a deletion of 13 amino acids. The extended hinge‐region of IgA1 adds additional flexibility to the Fab fragments and thereby increases the antigen‐binding ability of IgA1 molecules. The hinge‐region of human IgA1 is the only known substrate susceptible to the selective proteolytic cleavage by numerous and heterogeneous IgA1‐specific proteases produced by pathogenic bacteria such as *Streptococcus pneumoniae* , *Haemophilus influenzae* , *Neisseria gonorrhoeae* , *N. meningitidis* , and others [21]. Although the hinge region is present in IgA of other vertebrate species (e.g., cows, pigs, dogs, mice, and rabbits), it exhibits a low degree of sequence homology, glycosylation, and susceptibility to bacterial IgA1‐specific proteases, compared to human IgA1 [22]. Notably, IgA molecules of the above‐mentioned species structurally resemble human IgA2. IgA2‐like mucosal antibodies protect these species against invasion by certain bacteria. In humans, IgA2 plays a major role against infection by such bacteria due to its resistance to proteases, explaining the significant presence of IgA2 antibodies in mucosal secretions. It remains unknown, however, why humans have also significant amounts of secretory (S) IgA1 antibodies.

The hinge‐region is critical for IgA form and function. The hinge region of the α heavy chains is proline‐rich and, unlike IgG, does not have any disulfide bonds joining the two heavy chains. The main difference between IgA1 and IgA2 is the length and glycosylation of the hinge region. The IgA1 hinge region is 23 amino acids long, and rich in threonine/serine residues that are potential sites of O‐glycosylation, whereas the IgA2 hinge region is 13 amino acid residues shorter, and does not contain any potential O‐glycosylation sites [13]. While the longer hinge region gives greater flexibility to the Fab regions of IgA1, it also makes it more vulnerable to proteases made by bacteria such as *Neisseria meningitidis* , *Haemophilus influenzae,* and *Streptococcus pneumoniae* [13, 21]. While the longer hinge region for IgA1 is more flexible than IgA2 subclasses, both are less flexible than the IgG hinge region and tend to maintain a more flexed posture endowing a T‐shaped, rather than Y‐shaped, conformation [23]. This allows the Fab domains to reach across longer distances when crosslinking antigens [24].

#### 2.1.1 Dimeric and Polymeric IgA

Serum IgA originates primarily from bone marrow antibody‐secreting cells and is 85%–90% monomeric, while almost all (> 95%) of IgA secreted into mucosal tracts is in dimeric form. Dimeric and polymeric forms of IgA are also found in the circulation although in minor amounts (~10%). Both IgA1 and IgA2 subclasses are capable of forming dimeric and larger, polymeric molecules. As with IgM, the joining (J)‐chain is central to the formation of IgA multimers. The J‐chain is a 15 kDa polypeptide produced by antibody‐secreting plasma cells (PC) that is highly conserved across species [15], it has eight cysteine residues, six of which form internal bonds with each other, the remaining two facilitate dimeric linkages between IgA monomers. The penultimate cysteine on the tailpiece of a mIgA molecule forms a disulfide bond with the J‐chain, which in turn forms a disulfide bond with a second mIgA molecule [25]. Formation of higher order pIgA such as tetramers and pentamers then follows by the addition of further mIgA to this precursor via tailpiece‐to‐tailpiece disulfide bonds [26]. Liaisons between neighboring Cα2/Cα3 domains and hydrophobic side‐chains of Fc tail‐pieces forming a “molecular zipper” within the J‐chain scaffold of the founding dimer, give further support to larger polymers [27]. Dimers and polymers form in‐plane, and IgA pentamers adopt an asymmetric pentagon resembling IgM, with the largest gap being 50° [27, 28].

While serum IgA in humans is mostly monomeric, in mice serum IgA is predominantly dimeric [29].

#### 2.1.2 Secretion of IgA and Its Association With the Secretory Component (SC)

For dimeric IgA produced by antibody‐secreting cells (ASCs) in the lamina propria to pass onto the external mucosal surface, it must engage the polymeric immunoglobulin receptor (pIgR) at the basolateral surface of enterocytes in a J‐chain‐dependent manner [30, 31], before it is transcytosed to the apical side and secreted into the intestinal lumen [32]. pIgR has five extracellular Ig‐Doms (D1–D5, which fold over on themselves), a 23 amino acid transmembrane section, and an intracellular tail [33]. On contact with the JC in dIgA, D1 to D5 of pIgR unfold, exposing a D1–D3 interface which allows noncovalent binding of D1 with Cα2‐Cα3 on one IgA Fc region. The D1 of pIgR also interacts with the dimer's JC at its C‐terminus. This causes further conformational change at the other end of the pIgR extracellular domain, leading to the severance of Cys468's disulfide bond within D5 to Cys502, in exchange for a disulfide bond with Cys311 on the Cα2 domain of the second IgA Fc region, stabilizing the dimer [27]. Before secretion at the apical side, the extracellular portion (D1–D5) of pIgR, which is now wrapped around the Fc portions of the attached dIgA, is cleaved from the cell membrane by an unknown protease(s) and remains attached to the secretory IgA (SIgA) as the SC [34]. dIgA2m (1) is unable to form a covalent bond with the SC, making their association more vulnerable [35].

The SC has a molecular weight ranging from 50 to 80 kDa, depending on its glycosylation state [36]. A major role of the SC is to protect SIgA from proteases produced by microorganisms however, it is not impenetrable, but does delay degradation [37]. Indeed, Moon et al. have shown that the SC can be removed from SIgA in the intestine by commensal flora [38]. As well as in its bound form, to SIgA, SC also exists in a free state, and in both instances is capable of binding to bacteria such as *Streptococcus pneumoniae* and *E. coli* either through its peptide residues or interactions of its N‐glycans with bacterial lectins. The free form of SC can also neutralize *Clostridioides difficile* Toxin A [37]. The bacterial binding capacity of SC is a double‐edged sword, however, as it can be hijacked by bacteria to invade cells expressing the pIgR [34].

#### 2.1.3 IgA Glycosylation

All IgA is glycosylated, and glycans play important roles in its functions, including antigen binding and effector functions. IgA molecules and their related structures undergo considerable posttranslational modification by glycosylation, accounting for up to 10% of the molecular weight of IgA, 8% for the J chain, and 22% for the SC [13]. IgA1 can undergo both N‐ and O‐glycosylation, while IgA2 allotypes are only N‐glycosylated. Glycosylation of SIgA is much richer and more complex than that of serum IgA [39]. These posttranslational modifications have important consequences for form and function. Notably, they facilitate noncognate interactions with microorganisms and control the activation of complement.

##### 2.1.3.1 N‐Glycosylation of IgA

N‐linked glycans are added to the amide nitrogen of asparagine side‐chains. N‐glycans found on the heavy chains of human IgA are mainly complex, di‐galactosylated biantennary structures with a smaller proportion of triantennary branching [40, 41]. Despite amino acid sequences of IgA displaying major differences between mammals and birds, N‐linked glycans of the tailpiece are well conserved [14]. N‐glycosylation accounts for 6%–7% of the mass of human IgA1 and 8%–10% of the mass of human IgA2 [42]. Each of the IgA1 and IgA2 heavy chains is N‐glycosylated at Asn 263 in the Cα2 domain and Asn 459 at C‐terminal end of the tail‐piece. The IgA2 allotypes are further N‐glycosylated at Asn166 in Cα1 domain and Asn 337 of the Cα2 domain. A feature that differentiates IgA2m (2) and IgA2m(n) from IgA2m (1) is that they bear an additional 5th N‐glycan chain at Asn211 on the Cα1 domain of each heavy chain [11, 43]. These IgA2 residues generate proinflammatory responses involving neutrophils and macrophages suggesting a role of these glycans in inflammation probably following interaction with FcαRI [44, 45].

Important differences in N‐glycosylation exist between mIgA and pIgA. pIgA has more oligomannans and is less sialylated than mIgA, enhancing its capacity to activate the lectin complement pathway [46]. N‐linked glycans have been shown to control the formation of pIgA [47]. While the IgA1‐Fc Asn459‐glycan is needed for dimer formation, those at Asn263 stabilize the Fc‐J‐chain complex [48].

The J‐chain has one N‐glycosylation point at Asn48, which exists predominantly as a complex biantennary glycoform found to be sialylated in over 75% of measurements [40]. This N‐glycan seems essential for J‐chain function, as mutating Asn48 to alanine prevents the formation of IgA dimers [49].

The SC has seven sites of N‐glycosylation. Between the N‐glycosylation of the H_α_ chain, the J‐chain, and the SC, SIgA is well armored by a line of glycans in conformation [27]. N‐glycans on the heavy chains of IgA1 and IgA2 contain terminal GlcNAc and mannose residues that are normally concealed by the SC however, disruption of the noncovalent bonding between SC and the Fc regions of IgA1 or IgA2 causes them to be exposed, facilitating activation of the lectin complement pathway [50]. A minority of IgA Fab fragments have been reported to be N‐glycosylated however, their role is uncertain [40].

Besides the known abnormal O‐glycosylation of IgA1 in IgA nephropathy (IgAN), N‐glycosylation of IgA is also altered in this kidney disease. Dotz et al. have reported reduced galactosylation and increased bisection, fucosylation, and sialylation of IgA N‐glycans in patients with IgAN compared to healthy controls; N‐glycosylation of IgA also associated with the severity of renal failure [41].

##### 2.1.3.2 O‐Glycosylation

Mucins and soluble glycoproteins are typically O‐glycosylated by the transfer of N‐acetylgalactosamine (GalNAc) to an oxygen molecule on the side chain of either a threonine or serine residue. There are many enzymes capable of transferring GalNac. While there is a preference for threonine and serine residues in peptide sequences rich in proline and alanine residues, as yet, there is no predictable pattern of O‐glycoylation sites. Unlike N‐glycosylation, where glycan chains can be transferred *en bloc*, the extension of O‐glycan chains proceeds by the addition of individual saccharides [51].

Among human IgA, O‐glycosylation is restricted to the hinge‐region of IgA1. There are nine threonine and serine residues in the hinge region and it has been demonstrated that only 3–6 of them, on any given α1 heavy chain, are occupied by O‐glycans. The three core O‐glycosylated hinge‐region residues are Thr228, Ser230, and Ser232; Thr225, and Thr236 are occasionally glycosylated, while Thr233 is rarely glycosylated as a 6th residue [40, 52]. Ohyama et al. have shown in samples from healthy donors of African origin that, after treatment with neuraminidase to remove sialic acid and an O‐glycanase to remove GalNAc‐Gal (Core 1) residues (which will confound Neucα2‐6GalNAc residues for truly pathogenic deglycosylated residues), the most common galactose deficient site was Thr236, followed by Ser230, Thr233, Thr228, and Ser232 [53].

The process of O‐glycosylation of the IgA1 hinge‐region has been well characterized, given its importance in the pathophysiology of IgAN. The addition of GalNAc to a threonine/serine residue is catalyzed by UDP‐N‐acetylgalactosaminyltransferase 2 [54]. Sequential addition of galactose, to form the Core 1 structure, is mediated by β1‐3 galactosyltransferase (C1β3GalT1) which transfers galactose from a UDP‐galactose donor; this process is facilitated by a chaperone, Core‐1‐β3‐Gal‐T‐specific molecular chaperone (Cosmc) [55]. The core 1 can then undergo sialylation by α2‐6 linkage to GalNAc, mediated by the sialyltransferase ST6GalNAcII and/or additional α2‐3 sialylation of the galactose residue by a separate sialyltransferase. Sialic acid generally functions as a capping residue on O‐glycan chains so, alternatively, if α2‐6 sialylation of O‐GalNAc occurs before the transfer of galactose to the founding O‐GalNAc residue, the process terminates at that point [56, 57]. Proponents of the defective endogenous IgA1 production hypothesis for IgAN pathogenesis, argue that an imbalance between sialyltransferase and galactosyltransferase activity could lead to under galactosylation of IgA1 in IgAN [55]. However, this is premised on sialylation being normal or increased on IgA1 from IgAN patients, which is not the case [58]. While polymorphisms of *C1GALT1* encoding the galactosyltransferase C1β3GalT1 have been reported in Chinese patients with IgAN [59], this locus has not emerged in GWAS of IgAN.

## 3 IgA Synthesis

In healthy adults, the majority of human PC are committed to produce IgA, being by far the most abundant Ig in the body [60]. More IgA is produced per day (66 mg/kg/d) than all other Ig classes combined. At a concentration of about 2–3 mg/mL, IgA is the second most prevalent antibody in the serum after IgG [61]. This high synthesis rate is associated with a short half‐life of circulating IgA of about 3 days indicating the real need for this antibody. About one‐third of IgA is secreted directly into the vascular compartment and never reaches the mucosal surfaces [62]. Mucosal IgA is mostly generated at mucosa‐associated lymphoid tissues (MALT) including Peyer's patches located mostly in ileum, lamina propria, and surrounding lymph nodes, whereas circulating IgA comes mainly from bone marrow and spleen.

Since the 80's it has been questioned whether intravascular and mucosal IgA originate from two separate but related systems of immune defense [62]. There are, in fact, striking differences in the IgA system between systemic and mucosal sites. While serum IgA is mainly in monomeric form and belongs to IgA1 subclass, mucosal IgA is produced as dimers generated by the J‐chain insertion [63], belonging to both IgA1 and IgA2 subclasses in equivalent amounts.

The bone marrow is a primary lymphoid organ that not only acts as a nursery for hematopoietic stem cells but also “houses” terminally differentiated PC. In humans, despite IgG being the predominant serum antibody isotype, bone marrow contains a considerable number of IgA^+^ PC [64]. The fact that more than 80% of circulating IgA^+^ PC express α4β7 or CCR10 at steady state (a characteristic of PC generated in the GALT), implies that there is an interchange between cells of the mucosal and systemic compartments. It is likely thus that IgA^+^ PC primed in the gut can move to the bone marrow. The assumption that bone marrow‐derived IgA+ PC comes from the GALT is based on data showing that approximately 40% of these cells express CCR10 [64]. Interestingly, bone marrow IgA^+^ PC specific for microbial antigens is resistant to anti‐CD20 depletion treatment. As mucosal‐derived IgA+ PC do not express CD20 [65] on the contrary of IgG+ PC [66], these data suggest a stable contribution of mucosal‐derived PC to the bone marrow PC pool [67] and may explain the failure of rituximab trials in IgA‐associated diseases such as the one in IgAN [68].

In mucosal germinal centers, dimeric IgA is produced by PC at submucosa and transported from the basolateral epithelial compartment to the apical/luminal side by pIgR (vide supra). SIgA is prominently secreted at mucosal surfaces and coats a fraction of the commensal microbiota, a process that is critical for intestinal homeostasis [69]. IgA secretion limits the expansion of particular bacteria and regulates the microbial composition while, in turn, bacteria are capable of degrading SIgA, including removal of the SC [38, 70]. It remains, however, poorly understood how SIgA antibody production is controlled in mucosal lymphoid sites.

IgA originates from diverse B‐cell subsets under various conditions of T‐cell support. Human peripheral B cells include subsets of IgA^+^ B cells stratified as classical CD45RB(+)CD27(−) early memory population, a class‐switched CD39(+) tonsil‐resident population, CD19(hi)CD11c(+) memory population that potently responds to immune activation and IgA^+^ CD138^+^ PC [71]. IgA‐switched cells can be found among both T‐dependent (B2) and T‐independent (B1) cell subsets [72]. T‐cell‐dependent high‐affinity antibodies against protein antigens are typically generated during germinal center reactions between T‐follicular helper and follicular B cells [73]. Recently, it has been shown that a subset of innate‐like T lymphocytes, the mucosal‐associated invariant T (MAIT) cells [74], are capable of promoting IgA production by B cells [75, 76]. MAIT cells express a semi‐invariant TCR characterized by invariant TCRα made of Vα7.2 linked to Jα33, 12, or 20 in humans [77]. MAIT cells are conserved in mammals and mouse MAIT cells express Vα19‐Jα33 TCRα chain [74, 77]. MAIT cells are enriched in human blood, liver, and mucosa and are known for their ability to respond rapidly to microbial vitamin B metabolites presented on the MHC class I‐related protein MR1 [78]. MAIT cells thus recognize MR1‐presented antigens derived from microbial riboflavin biosynthesis and mount protective immune responses against microbes producing such metabolites. Upon stimulation, MAIT cells produce proinflammatory cytokines, including IFNγ, TNFα, and IL‐17A, and cytotoxic molecules, including granzyme B and perforin [79, 80, 81]. Using MAIT‐deficient mice (MR1^−/−^), several studies have demonstrated their role in immunity against mucosal bacterial pathogens [82, 83, 84, 85]. Of note, *S. pyogenes or pneumoniae* and *S. aureus* are able to activate MAIT cells through their ability to produce a MAIT cell ligand, derived from the riboflavin synthesis pathway, or by secreting the toxin leukocidin ED, respectively [86, 87, 88, 89]. Although MAIT cells have originally been described as abundant in mucosal sites little is known about the role of these cells in mucosal immunity, notably in SIgA production. MAIT cells support B‐cell response in the context of infection. Upon virus (SIV) and bacterial (Vibrio cholera) mucosal infections, MAIT‐cell abundance is associated with mucosal IgA production [76, 90]. Moreover, MAIT cells can directly activate B‐cell maturation and antibody production. It has recently been shown that MAIT cells activate dendritic cells (DC) to promote T(Fh)‐cell differentiation and induce humoral systemic immunity [91, 92].

### 3.1 B‐Cell Class Switching to IgA: Role of Cytokines and Chemokines

The lymphoid microenvironment influences IgA PC production notably via several soluble factors released in effector niches. Cytokines and chemokines play a crucial role in the development of IgA^+^ PC [93]. In adults, it is estimated that ~80% of all PC are found in the intestinal lamina propria. In the duodenum about three‐quarters of these are IgA^+^, while in the colon IgA^+^ predominance increases to 90% of PCs [94]. Most other intestinal PCs secrete IgM [95]. Immunoglobulins delivered in maternal milk inhibit the development of GALT [96] and, in humans, the mucosal IgA response is delayed until after 1 month of age due to reduced expression of TACI and its receptors [97].

Maturation of naïve GALT B cells into IgA‐secreting plasmablasts and PC, collectively termed ASCs, can be stimulated by both T‐cell‐dependent and T‐cell‐independent mechanisms. Classic T‐cell‐dependent mechanisms are mediated through MHCII presentation of antigen by CD4^+^ T cells to B cells in lymphoid tissue with CD40‐CD40L co‐stimulation and IL‐10, TGF‐β cytokines. However, this T‐cell‐dependent mechanism takes 5–7 days to develop, which is too slow for imminent threats [98]. The intestinal mucosal environment is comprehensively pro‐IgA, consolidated by TSLP secretion by the epithelium and microbiota triggering of NO release by Tip DCs, which increases B‐cell TGF‐bRII expression, making the role of helper T cells dispensable for IgA class‐switching and maturation [98, 99, 100]. Mora et al. have described T‐cell‐independent IgA class‐switching and maturation of B cells mediated by intestinal DCs. RA production by intestinal DCs was enough to imprint a gut‐homing phenotype on B cells, and synergism with IL‐6 or IL‐5 led to concurrent IgA class switching [101]. Tezuka et al. have shown the plasmacytoid DCs promote B cells to IgA^+^ PCs through secreting APRIL and BAFF, independently of T cells [102]. The ability of mucosal DCs to convert vitamin A to RA with retinaldehyde dehydrogenase type 2 (RALDH2) is key to T‐cell‐independent IgA B‐cell class switching. This enzyme is expressed by CD103^+^CD11b^+^ DCs, Tip DCs, and TLR5^+^ DCs in the intestinal mucosa [99]. Similar to T cells, this exposure to RA upregulates the expression of a4b7 and CCR9 on B‐cell plasmablasts, which ensures homing to the intestine. Plasmablasts generated in cecal lymphoid tissue also express CCR10, which directs them back to the large intestine through CCL28 recognition [103].

In mice, it has been demonstrated that commensal bacteria present in both the small intestine and the large intestine are coated with IgA, whereas commensals unique to the colon are not coated in IgA [104]. This is consistent with the concentration of GALT in the small intestine, and the dominance of more benign anaerobic bacteria in the colon. In humans, both IgA1 and IgA2 isotypes are secreted in the intestine in similar measures however, in the small intestine there is about a 60% IgA1 predominance and in the large intestine it is IgA2 that has about 60% predominance [105, 106]. Protein antigens tend to generate an IgA1 response while polysaccharide antigens induce an IgA2 response [107]. In patients with small intestinal bacterial overgrowth (SIBO), the proportion of IgA2 class switched ASCs increases [105]. This could be to employ IgA2's greater resistance to bacterial proteases.

The antibody repertoire of IgA^+^ ASCs in the mucosa is restricted compared to systemic immune responses, and the genetic variability seen in the V_H_ complementarity determining region 3 (CDR3) is more consistent with somatic mutations, rather than the clonal evolution patterns that result from affinity maturation in germinal centers [108]. This could reflect a high‐level of T‐cell‐independent IgA production and suggests a system that relies more on noncognate, innate, binding of SIgA to bacteria mediated by glycosylation [50], over refined CDR specificity. Using germ‐free mice exposed to defined populations of bacteria by either the mucosal or systemic route, Li et al. also demonstrated restricted heterogeneity in the mucosal IgA response and found that the threshold of bacteria required to influence the immunoglobulin response was much higher for mucosal exposures than for systemic exposures [109]. Unsurprisingly, mucosal exposure promoted IgA class switching while systemic exposure promoted IgG class switching. Remarkably, increasing the mucosal burden of bacterial exposure reduced the IgA^+^ PC repertoire, while the converse effect of systemic exposure was seen on IgG^+^ PC repertoire. Importantly, the overlap between the two immune compartments was seen, with small systemic IgG responses seen in response to mucosal exposure and small SIgA responses seen on systemic exposure [109].

In the gut, most IgA^+^ PC are found in the lamina propria. IgA^+^ B cells generated in the GALT and mesenteric lymph nodes display gut‐homing phenotype through the expression of CCR9, CCR10, and α4β7 molecules. IgA^+^ PC then migrates out of the efferent lymphatics, via the thoracic duct, entering into the gut lamina propria through MAdCAM‐1 interaction on endothelium [69]. Recently, it has been shown that MZB1 regulates IgA generation by PC in the gut and defends the mucosal barrier during inflammation [110]. MZB1 is an endoplasmic reticulum (ER)‐localized protein constitutively expressed in innate‐like B cells, such as marginal zone (MZ) B cells and B1 cells, and highly upregulated during plasma cell differentiation. In the ER, MZB1 associates with antibody heavy and light chains (HC and LC, respectively) to promote the assembly and secretion of pIgA. Thus, MZB1 functions as a molecular chaperone facilitating the formation of polymeric IgA, especially during rapid responses. Interestingly, ER stress in intestinal epithelial cells has been also shown by others to be able to induce a polyreactive IgA response, which was protective against enteric inflammation [111].

Similar to the bone marrow, APRIL and IL‐6 are cytokines that promote the survival of IgA+ PC in the gut lamina propria [112]. Eosinophils, a leukocyte commonly found in the gut, are also implicated in the IgA^+^ PC survival in lamina propria [113]. Eosinophil depletion resulted in the reduction in IgA^+^ PC and altered microbiota, indicating that their ability to secrete APRIL seems to be crucial to establish IgA^+^ PC and maintaining intestinal homeostasis [114].

The longevity of human IgA^+^ PC in the gut lamina propria is remarkable, with cells surviving up to 22 years [115]. Similarly in mice, nonproliferative PC were detected up to 9 months following oral immunization with cholera toxin and ovalbumin [116].

## 4 Physiologic Roles of IgA at Systemic and Mucosal Sites

IgA is differentially distributed between the systemic and mucosal immune systems and plays a key role in immune defense [117, 118]. SIgA plays an important role in different functions in the mucosal immune system. While high‐affinity IgA antibodies (from T‐cell‐dependent pathways) are thought to protect intestinal mucosal surfaces against invasion by pathogenic microorganisms, low‐affinity IgA antibodies (from T‐cell‐independent pathways) are important for confining commensal bacteria to the intestinal lumen [60]. Serum monomeric IgA (mIgA) is thought to play a minor role in systemic immune responses. The major role of serum mIgA in physiology is to promote anti‐inflammatory effects. It has been demonstrated by several groups more than 30 years ago that serum IgA is capable of downregulating many cell responses [8]. However, the molecular basis for such an action remained elusive until the discovery of FcαRI (CD89)'s ability to mediate inhibition through the ITAM of their associated FcRγ chain. IgA is classically known for neutralizing toxins and bacteria (viruses) at mucosal surfaces [119, 120] by interfering with their motility, competing for epithelial adhesion sites, and improving the viscoelastic properties of the airway secretions [121]. The SC not only protects SIgA from proteolytic degradation but is also involved in establishing local interactions with bronchial mucus, thereby contributing to the “trapping” and removal of the antigen (“immune exclusion”) [122].

### 4.1 Anti‐Inflammatory Functions of the mIgA Molecule

The ligation of Ig‐complexed antigen to Fc receptors (FcR) can trigger numerous cellular effector functions including phagocytosis, antibody‐dependent cellular cytotoxicity (ADCC), and the secretion of cytokines or other inflammatory mediators [123]. These crucial activating functions are essential to protect the host against infections by linking the humoral and cellular arms of immune responses. FcR activating functions depend on the receptor's association with ITAM‐bearing adaptors, such as FcRγ or FcRβ chains.

FcαRI is an IgA Fc receptor expressed by myeloid cells which binds both IgA subclasses. It is associated with the ITAM‐bearing adaptor FcRγ but not the FcRβ chain. The FcRγ‐chain ITAM consists of a conserved stretch of paired tyrosines and leucines separated by seven amino acids in a consensus sequence (YxxLx_6‐7_YxxL) [124]. The *Src* kinase phosphorylates the tyrosines within the associated FcRγ ITAM. These then serve as “docking” sites for the recruitment of the tyrosine kinase Syk, which facilitates the activation of multiple targets, such as the PI3K, and induces the downstream release of IP3 and diacylglycerol to trigger calcium release, the activation of Raf‐1—MEK—MAP kinases signaling pathways and subsequently cell activation [125].

**FIGURE 1:**
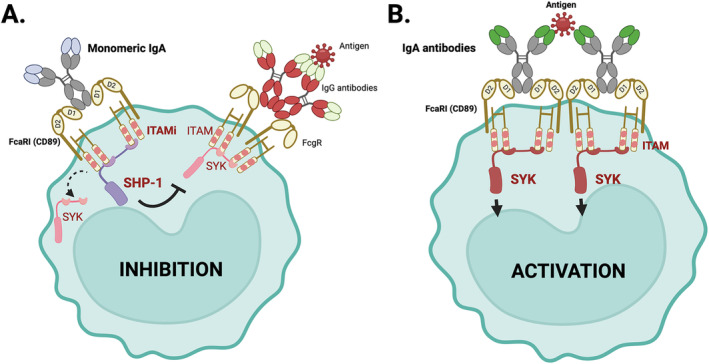
Inhibitory and activating ITAM signaling indices by IgA binding to its receptor, the FcαRI (CD89). (A) Monomeric IgA (free of antigen) binds to two FcαRI molecules resulting in weak phosphorylation of immunoreceptor tyrosine‐based activation motifs leading inhibitory ITAM (ITAMi) configuration with recruitment of Src homology region 2 domain‐containing phosphatase‐1 (SHP‐1). This results in inhibition of ITAM signaling of heterologous receptors (i.e., IgG‐mediated FcγR ITAM signaling), and impairs phosphorylation of spleen tyrosine kinase (Syk), LAT, and ERK. (B) IgA antibodies bound to an antigen induce cross‐linking of at least four FcαRI molecules, resulting in sustained ITAM phosphorylation of the associated FcR γ‐chain. Phosphorylated ITAMs subsequently function as a docking site for signaling molecules such as Syk. Syk plays an essential role in initiating signaling pathways, including the Ras/Raf/MEK/MAPK pathway. Activation of signaling pathways results in proinflammatory responses such as phagocytosis, antibody‐dependent cellular cytotoxicity, respiratory burst, degranulation, antigen presentation, and release of cytokines, inflammatory mediators, and NETs. Created with BioRender.com.

Interestingly, FcαRI can act as a bifunctional receptor [126, 127] such that different receptor signaling can either activate or inhibit a heterogeneous signal depending on whether the IgA ligand is monomeric or polymeric. While polymeric IgA or IgA immune complexes lead to strong receptor aggregation and stable recruitment of Syk kinase, mIgA that cross‐links two FcαRI receptors, as described from the crystal structure studies [128, 129], induces only transiently recruitment of Syk which is followed by stable and prolonged SHP‐1 phosphatase recruitment. The interaction of the CH2/CH3 Fc section of each IgA with the D1 domains of each receptor is weak, but essential to induce tyrosine phosphorylation of the ITAMs leading to an inhibitory ITAM configuration (ITAMi) (Figure 1A). The 1:2 ratio between IgA:FcαRI is able to induce ITAMi signals as confirmed by using F(ab')_2_ of monoclonal antibodies to cross‐link pairs of FcαRI [126]. The monovalent effect of Fab anti‐FcαRI alone may be explained by a population of FcαRI expressed at the cell surface as dimers, as observed on U937 cells following surface chemical crosslinking experiments (Monteiro RC, unpublished). mIgA binds with low affinity to the FcαRI but enough to activate the ITAMi signaling [126] inhibiting the activation of heterologous receptors (such as other FcR, cytokine receptors, chemokine receptors, and TLRs) [126, 130].

First evidences of ITAM‐bearing receptor bi‐functionality was shown by recruitment of SHP‐1 and SHIP following activation of FcεRI, FcγRIIa, and TCR [131, 132, 133]. In the case of FcεRI recruitment of the SH2 domain phosphatidylinositol phosphatases SHIP1/2 following its hyperaggregation induced by an excess of ligand abrogates mast cell activation [134] further indicating general property of ITAM‐bearing receptors to be bi‐functional, depending on the type or quantity of their ligands. However, in all these cases, phosphatase recruitment by ITAM‐associated receptors was thought to mediate a negative feedback loop regulating activation signals induced by their own receptors.

**FIGURE 2:**
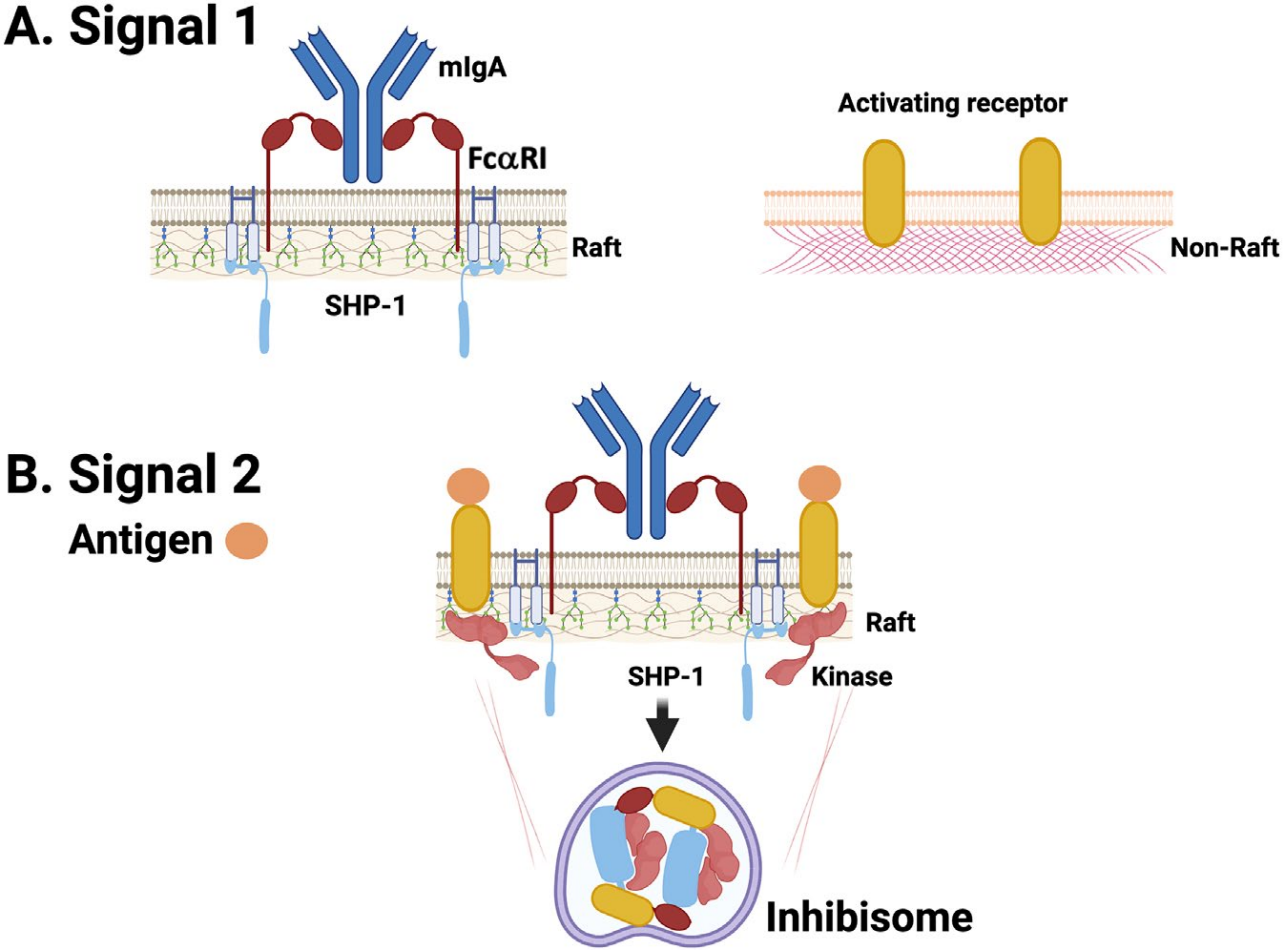
Mechanism of ITAMi signaling pathway in the absence of receptor‐induced coaggregation with heterologous receptor in the cell surface. During resting conditions, the FcaRI expressed by myeloid cells constitutively binds circulating monomeric IgA which leads to its association with SHP‐1 in lipid rafts creating an ITAMi signaling configuration. After an inflammatory signal induced by a given antigen, an activating receptor is engaged into raft domains, before being internalized together with FcaRI under the inhibitory ITAM configuration which is armed of SHP‐1 and segregated into polarized clusters named inhibisomes. These structures enable a close proximity between FcaRI‐bound SHP‐1 to the antigen‐bound receptor allowing sustained inhibition of the receptor‐activated kinases. Created with BioRender.com.

Another particularity of ITAMi signaling is that after the recruitment of tyrosine phosphatase‐1 (SHP‐1) to the FcRγ ITAM and the movement of FcαRI to lipid rafts both inhibitory and activating receptors and the inhibitory molecular effector (SHP‐1) can be found in intracellular clusters that we have named “inhibisomes” [135] (Figure 2). These intracellular clusters are essential to promote inhibition of different signaling pathways induced by the heterologous receptors without initial membrane receptor aggregation and differing from ITIM‐bearing FcγRIIB in which coaggregates were largely excluded from raft localization staying in the cell surface plasma membrane [135]. Despite these differences, it is interesting to note that FcγRIIB inhibition affects not only signals triggered by FcγRIIB‐engaged activating receptors in a cis‐inhibitory mode but also signals triggered by receptors engaged independently in a trans‐inhibitory mode [136]. This brings some similarities between both types of inhibitory immunoreceptors acting at a distance without co‐aggregation with heterologous activating receptors. There are however not only differences in cellular localization but also in the action time between these two types of inhibitory receptors. Although ITAMi has a long‐lasting inhibitory effect (~12 h), it takes some time to start inhibition due to a Syk‐dependent preactivation phase that precedes SHP‐1 recruitment [126], whereas ITIM is immediately operational by recruiting phosphatases just after receptor co‐engagement [124].

Although initial ITAMi data were obtained mostly with Fab or F(ab')_2_ fragments of anti‐FcαRI mAbs, latter experiments done with highly purified plasma‐derived human mIgA (> 99% of purity) produced at CSL Behring (Bern, Switzerland) showed inhibition of multiple heterologous receptors as evaluated on FcαRI+ cell transfectants, blood phagocytes from healthy individuals, and synovial cells from RA patients [137]. FcαRI‐transgenic mice and wild‐type mice treated with mIgA were studied in models of collagen antibody‐induced arthritis (CAIA) and collagen‐induced arthritis (CIA). The mice were assessed for the development of arthritis using an arthritis score, and joint tissue samples were evaluated for the extent of leukocyte infiltration and expression of phosphatase.

Treatment with human mIgA not only impaired cell activation in an FcαRI‐FcRγ‐dependent manner with an ITAMi signature but also human mIgA was strongly effective in either preventing or attenuating CAIA or CIA in FcαRI‐transgenic mice. Administration of mIgA markedly inhibited the recruitment of leukocytes to the inflamed joints of mice, which was associated with the induction of SHP‐1 phosphorylation in joint tissue cells. Moreover, mIgA reversed the state of inflammation in the synovial fluid of RA patients by inducing an ITAMi configuration [137].

Although ITAMi induction mechanism remains partially understood, the protein kinases Lyn and Fyn were shown to play alternative roles in immunoreceptor function [138]. While SHP‐1 tyrosine 536 phosphorylation by Lyn activates the phosphatase promoting inhibitory signaling through the immunoreceptor, Fyn‐dependent phosphorylation of SHP‐1 serine 591 inactivates the phosphatase, enabling activation of a given immunoreceptor. This was demonstrated in vivo in mice, where Lyn deficiency exacerbates nephritis and arthritis, whereas Fyn deficiency is protective. Moreover, Fyn‐activating signature is detected in patients with lupus nephritis, highlighting the importance of this Lyn‐Fyn balance. These data bring light on understanding how receptors discriminate between negative and positive signals. ITAMi signals have been also described in other FcRs, such as CD16A and CD32A, as well as in TREM2 via DAP10 and in the BCR via CD79a [138, 139].

### 4.2 Homeostatic and Tolerogenic Roles of SIgA IgA Antibodies

In mucosa, SIgA is the principal immunoglobulin form found at epithelial surfaces. SIgA is produced by the binding of dimeric IgA to the pIgR at the basolateral side of the epithelium, which is transported to the luminal side by transcytosis. IgA is then released at the mucosal surface (lumen) by cleavage from the pIgR. During this process, the extracellular domain of the pIgR is proteolytically digested, generating the so‐called SC that remains attached to the IgA dimers, and together they form the SIgA molecule. SIgA promotes multiple functions such as immune exclusion by entrapping dietary antigens and microorganisms in the mucus, downregulates the expression of proinflammatory bacterial epitopes on commensal bacteria, and, in general, promotes the maintenance of appropriate bacterial communities within specific intestinal segments [140, 141]. In addition, SIgA blocks or sterically hinders microbial components involved in epithelial attachment, mediates intraepithelial neutralization of incoming pathogens and microbial inflammatory products, and facilitates antigen sampling by binding to microfold (M) cells, an epithelial‐like cell type specialized in antigen capturing [142, 143, 144].

SIgA plays a crucial role in microbiota‐host‐derived homeostasis [145, 146]. During the last two decades, SIgA has been redefined as a regulator of bacterial networks. Innate SIgA antibodies with polyreactive specificities are selected into the IgA repertoire upon recirculation in Peyer's patches [147]. This selection process occurs independently of microbiota or dietary antigens indicating that environmental factors may not influence IgA reactivity despite some SIgA acquired somatic mutations. These results suggest endogenous mechanisms driving the homeostatic production of polyreactive IgAs with innate specificity for the microbiota. Moreover, the identification of SIgA‐coated bacterial repertoires in gut microbiota highlights the important role of SIgA antibodies.

Donaldson et al. have shown that SIgA facilitates gut colonization by *B. fragilis* [70]. IgA has also been shown to support bacterial symbiosis by facilitating or modifying bacterial networks, implying that some bacteria may depend on other bacteria to persist in the community [148]. Studies of microbiota in SIgA‐deficient hospitalized patients revealed that bacterial networks are altered in the absence of IgA [149]. However, to eliminate confounding factors linked to hospitalized patients, further studies in asymptomatic SigA‐deficient individuals are needed to clearly understand the physiological role of SIgA‐coated microbiota in human health. The role of IgA‐coated bacteria and health was suggested by studies of microbiota of inflammatory bowel diseases [150]. Human IgA+ microbiota transferred to murine colitis models have shown that intestinal bacteria selected on the basis of high‐coating with IgA conferred susceptibility to colitis in germ‐free mice. In addition, patients treated with anti‐TNF‐alpha therapies exhibited altered microbiota‐specific IgA responses with increased IgA coating of *Oscillospira spp*., associated with a delay in time to surgery [151]. Moreover, IgA‐coated bacteria from the gut of children at 12 months of age seem associated with later development of allergic asthma [152]. Moreover, IgA‐coated bacteria are reduced in multiple sclerosis patients during disease relapse [153]. In an experimental mouse model of MS, the EAE model, the authors demonstrated that the removal of plasmablasts and PC resulted in exacerbated disease that was corrected by the administration of gut‐derived IgA+ PC. Finally, it was demonstrated in these animals that IgA‐secreting cells travel from the gut to the brain to attenuate neuroinflammation in an IL‐10‐dependent manner [153].

IgA response to symbiotic bacteria was shown to be a major mediator of gut homeostasis [154]. Specific IgA selects against a specific phase‐variable capsular polysaccharide is associated with reduced intestinal innate immune activation [141]. Interestingly, IgA antibodies seem to control Segmented Filamentous Bacteria growth indicating its role in pathobiont regulation. Indeed, AID−/− mice display SFB overgrowth and intestinal inflammation [155]. Together, these results suggest that selective IgA modulates bacterial fitness toward a noninflammatory function.

SIgA Fc domains have also an important function through their interaction with cells in mucosal secretions. For example, colostral phagocytes expressing FcαRI are continuously occupied by SIgA [156]. FcαR in colostrum neutrophils has a low or absent association with FcRγ chain, suggesting a neutrophil‐mediated anti‐inflammatory role of SIgA, which remains poorly understood [156]. These studies highlight the importance of long‐term breastfeeding during early life. In humans, mucosal SIgA in the fetal intestine is absent or rarely present until 10 days after birth [157].

**FIGURE 3:**
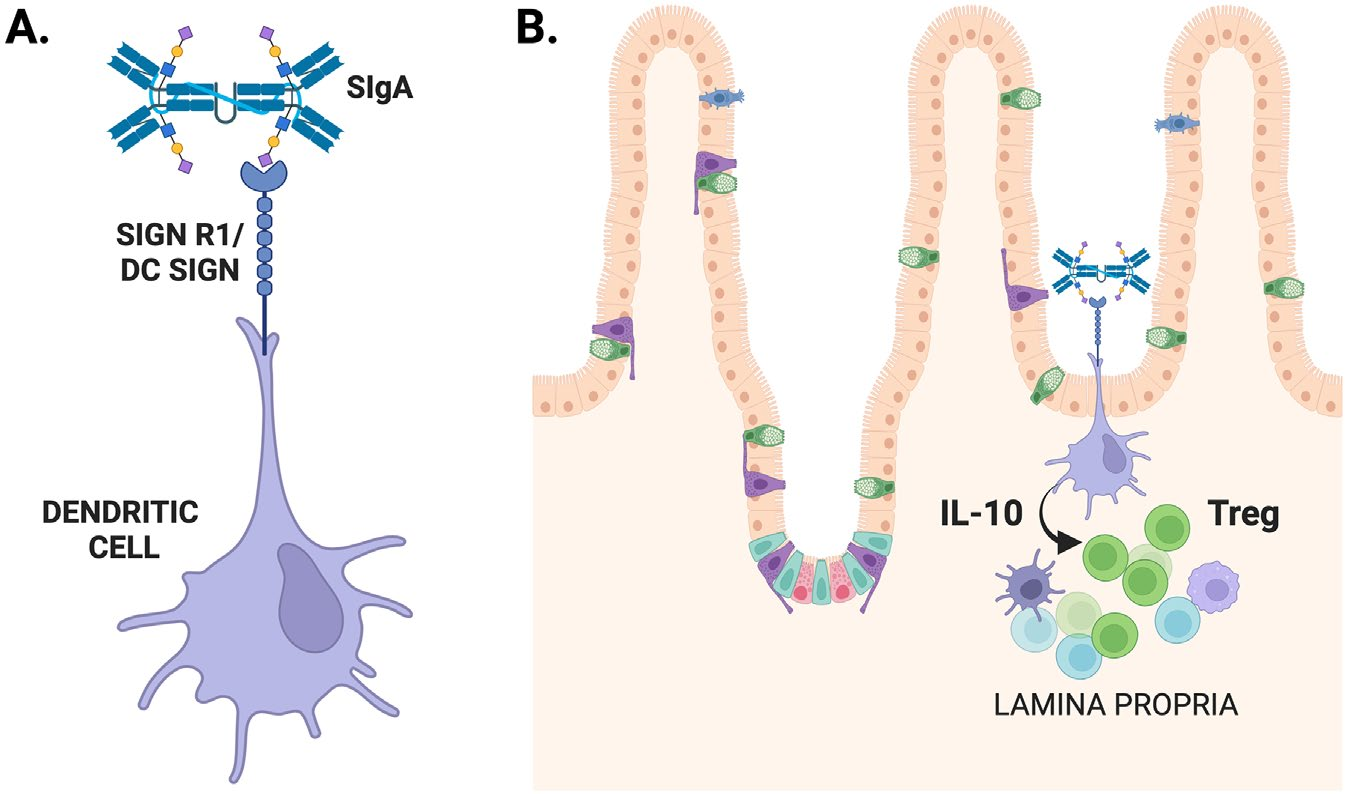
Tolerogenic role of secretory IgA interaction with SIGNR1 by dendritic cells. (A) Schematic representation of secretory IgA (SIgA) binding to SIGNR1. SIGNR1 is a mouse type II transmembrane protein with a short aminoterminal cytoplasmic tail, a neck region, and a single carboxyl terminal carbohydrate recognition domain (CRD; or C‐type lectin domain), and is expressed on immature monocyte‐derived DCs. SIGNR1 and DC‐SIGN recognize both internal branched mannose residues as well as terminal di‐mannoses, α1‐3 and α1‐4 fucosylated glycan structures, and certain N‐aceltylglucosamine containing molecules on self‐proteins and/or pathogens. SIgA‐SIGNR1 interaction depends on mannose residues and the presence of secretory components. (B) SIgA‐DCs generate the expansion of regulatory T cells through IL‐10 production. SIgA‐DCs are stronger inhibitors of autoimmune responses in mouse models of type 1 diabetes and multiple sclerosis indicating a SIgA‐dependent DC immunoregulation in mucosal sites. Created with BioRender.com.

SIgA has also a powerful anti‐inflammatory effect due to its ability to interact with DC through the SIGNR1 receptor [158]. SIGNR1 is a mouse homolog of DC‐SIGN, a C‐type lectin receptor that was recently described as a receptor for human SIgA on the cell surface of DC through carbohydrate‐recognizing receptors [159]. Similar to its interaction with DC‐SIGN, SIgA‐SIGNR1 interaction is dependent on sugars, notably mannose residues, and the presence of the SC [158] (Figure 3A). SIgA‐SIGNR1 interaction prevents the activation of the immune responses by different agents. SIgA inhibits the maturation and the production of proinflammatory cytokines by DC, which instead harbors a tolerogenic phenotype and produces large amounts of IL‐10. Importantly, SIgA‐DC interaction promotes the expansion of IL‐10‐secreting Foxp3^+^ Treg cells (Figure 3B), which prevents the development of autoimmune diseases in mice, such as experimental autoimmune encephalomyelitis and type 1 diabetes. Of note, triggering of DC‐SIGN by anti–DC‐SIGN fusion proteins can also induce DC tolerogenic activity through IL‐10 secretion [158, 160]. Thus, SIgA interaction with lectin‐like receptors on DCs, such as SIGNR1/DC‐SIGN may have major regulatory functions at mucosal and systemic levels.

### 4.3 Retro‐Transcytosis of Mucosal IgA Antibodies From Intestinal Lumen

To facilitate robust immune surveillance while maintaining the integrity of the intestinal barrier, the mucosal immune system has evolved a number of mechanisms to discretely sample antigens from the lumen. Lymphoid structures of the intestinal mucosa such as Peyer's patches in the distal ileum and isolated lymphoid follicles (ILFs) throughout the appendix, colon, and rectum, are overlaid by a specialized type of epithelium called follicle‐associated epithelium (FAE), designed to deliver antigen to the underlying lymphoid tissue. Approximately 10% of cells in the FAE are microfold cells (M‐cells); these are specialized phagocytic epithelial cells [161]. Depending on the route of sampling, a tolerant or more aggressive immune response will be curated against the sampled antigen. Procurement via M‐cells promotes a tolerogenic IgA immune response [69] while delivery through goblet cell‐associated antigen passages (GAPs) induces T‐regulatory cells [162].

A series of investigations have indicated that M‐cells can sample luminal contents by binding and endocytosing IgA‐bound antigens. Initial observations that IgA accumulates on the surface of intestinal M‐cells overlying Peyer's patches [163, 164] led to further experiments investigating its functional significance. Wetzin et al. found that exogenous administration of colloidal gold particles coated in IgA adhered exclusively to M‐cells, where they were endocytosed and transited retrogradely to the epithelial pocket invaginations on the basolateral side of M‐cells [165]. Mantis et al. later showed that this process was specific to IgA however, in mice at least, it was only observed for human colostral SIgA and human IgA2, as the hinge region of human IgA1 interfered with the process [166]. It is not clear if the SC permits attachment of both human IgA1 and IgA2 or if binding remains restricted to IgA2 in the presence of SC. Following this, Kadoui and Corthesy demonstrated that large IgA‐immune complexes, containing SIgA bound to *Shigella flexneri* , could be transferred to DCs in the subepithelial dome of Peyer's patches after enteral delivery. They showed that only CD11c^+^CD11b^+^ DCs isolated from a mucosal environment could internalize M‐cell delivered antigen, whereas CD11c^+^CD19^+^ DCs could bind, but not process, SIgA while CD11c^+^CD8α^+^ DCs did not engage the immune complexes at all [143]. It was later shown that these mucosal DCs bind retro‐transcytosed IgA via their DC‐SIGN receptor [167].

The mechanism of IgA2‐mediated retro‐transcytosis in M‐cells was not forthcoming. Heavy chain exchange experiments by Mantis et al. showed that both Ca1 and Ca2 domains were necessary for the binding of human IgA2 to murine M‐cells, excluding the role of FcaRI (CD89) which binds along the Ca2 and Ca3 domains. Removal of the human IgA1 hinge region conferred it with the ability to bind murine M‐cells, suggesting steric hindrance of this O‐glycosylated region with the unknown receptor [166].

Using an ingenious in vitro model of M‐cells, where human CaCo‐2 cells grown on inverted trans‐well platforms are put in co‐culture with subjacent Raji B cells, Rochereau et al. again demonstrated retro‐transcytosis of human IgA2, but not IgA1, and identified Dectin‐1 (a C‐type lectin PRR) and Siglec‐5 as co‐receptors for human IgA2 [167]. They showed that binding Cα1 domain alone, and that glycosylation, particularly sialylation, was necessary. Dependence on the Cα1 domain makes sense, as the SC interacts with the Cα2 and Cα3 domains [13]. Constructs of human IgA2 lacking N‐glycosylation, treated with an N‐glycosidase or with a sialidase showed significantly reduced retrotranscytosis [167]. Interestingly, CARD9, a genetic locus associated with IgAN [168], is involved in Dectin‐1 downstream signaling [169]. Another PRR, NOD2, which is a risk locus for inflammatory bowel disease, downregulates Dectin‐1 expression on M‐cells—patients with the risk locus demonstrate increased uptake of IgA2 in Peyer's patches [170].

Retro‐transcytosis of human IgA from the intestinal lumen, independently of M‐cells, has also been described. The transferrin receptor (CD71; TfR), commonly expressed on IECs, has previously been identified as a receptor of IgA1 [171]. In celiac disease, patients develop an inflammatory response to gliadin, a product of gluten, in the upper small intestine. On duodenal biopsies, these patients were found to have upregulated expression of CD71 on IECs. Matysiak‐Budnik et al. demonstrated that SIgA colocalized with CD71 at the apical pole of these enterocytes and, with Ussing chambers, showed that retro‐transcytosis of SIgA‐gliadin complexes is mediated by CD71 [172]. Later work using FRET proved interaction between CD71 and IgA in duodenal biopsies from patients with celiac disease and, also implicated transglutaminase‐2. Co‐immunoprecipitation using CaCO‐2 cells, a line of human colon cancer cells, confirmed interactions between SIgA, CD71, and transglutaminase‐2. Cellular biology studies showed that SIgA taken up by CD71 at the apical surface could pass through classic CD71 recycling endosomes, co‐localizing with EEA1, and be trafficked to the basolateral side of the cell, avoiding lysosomal degradation. While the SIgA used in these studies was not explicitly defined as IgA1 or IgA2, interactions were inhibited with pIgA1 saturation. Knockdown of CD71 and saturation with sCD71 incompletely reduced total IgA binding, possibly suggesting only the SIgA1 moiety of the experimental SIgA was inhibited and that SIgA2 was unhindered [173].

### 4.4 Proinflammatory Functions of the IgA Antibodies in the Circulation

IgA antibodies play an important role in host–pathogen defense by activating lymphocytes and myeloid cells through their interaction with receptors, such as FCLR3 or 4, and FcαRI (CD89) respectively [7, 174, 175]. Depending on the molecular form of IgA antibodies, proinflammatory responses can be modulated toward higher responses. It is well known that pathogen‐specific mucosal and systemic IgA antibodies following vaccination increase protective immunity against infectious diseases. For example, SIgA, which is predominant in human colostrum, plays an important role in protecting newborn infants against acute gastrointestinal and respiratory infections [176]. Colostral mononuclear phagocytes opsonized by SIgA are able to perform bacterial‐killing activity [177].

FcαRI (CD89) has also been described as an innate receptor, capable of directly binding bacteria independently of its cognate ligands, IgA, and C‐reactive protein (CRP) [7, 178, 179]. This binding is only partially inhibited by IgA and is able to induce bacterial phagocytosis by DC, monocytes, and macrophages, suggesting a role in innate host defense [180]. These observations were verified on cells from IgA‐deficient individuals in which blood phagocytes bind, internalize, and kill bacteria in a FcαRI‐dependent manner as well as in FcαRI‐transgenic mice that were protected from experimental sepsis [180]. Together, FcαRI emerges as a new class of innate receptors for various bacteria representing a first‐line of defense before adaptive responses can be mounted [180].

Other than phagocytosis, interaction of antigen‐bound IgA‐antibodies with FcαRI induces receptor aggregation leading to down‐stream activation of effector functions through SYK kinase recruitment (Figure 1B), such as degranulation, superoxide generation, release of neutrophil extracellular traps, ADCC, release of cytokines and chemokines, or antigen presentation [181]. FcαRI cross‐linking by IgA‐immune complexes on immature DCs induces antigen presentation through the major histocompatibility complex class II pathway, DC maturation, and production of IL‐ 10 [182, 183, 184]. FcαRI^+^DCs are also able to induce IgA^+^B cells in secondary lymphoid organs due to receptor‐mediated release of IL‐10 and TGF‐β which results in IgA isotype switching in B cells [185]. Interestingly, FcαRI triggering by IgA converts human intestinal CD103^+^ DCs into proinflammatory cells through glycolytic reprogramming resulting in the release of multiple cytokines such as TNF‐α, IL‐ 1β, IL‐6, and IL‐23 [186].

Recent data provide evidence for another IgA receptor specific to SIgA, the Fc‐like receptor 3 (FCLR3) [187]. FCRL3 expressed on Tregs binds to SIgA, which blocks their suppressive function. Moreover, FCRL3 stimulation on Tregs induced IL‐17, IL‐26, and IFNγ production and promotes expression of the Th17‐defining transcription factor RORγt without affecting FOXP3 expression. SIgA may thus act in the mucosa by locally modifying regulatory T‐cell plasticity to help control infection. Polymorphisms of the FCRL3 promoter, which alters gene expression, are associated with the risk of autoimmune diseases such as IgAN [188], revealing its physiologic importance [189].

## 5 Pathological Roles of IgA

The presence of altered IgA or excessive IgA‐immune complexes (IgA‐IC) can result in tissue injury and contribute to disease development. The persistence of circulating IgA‐IC continuously activates receptors, such as FcαRI on myeloid cells or CD71 on enterocytes/mesangial cells, resulting in tissue inflammation and organ damage. Several inflammatory and autoimmune diseases are associated with increased serum levels of IgA and/or IgA‐IC [117, 190]. These disorders include IgAN, Henoch‐Schönlein purpura (HSP), ankylosing spondylitis, Sjögren's syndrome, alcoholic liver cirrhosis, celiac disease, inflammatory bowel disease, and dermatitis herpetiformis [125, 191].

IgAN is the most common IgA‐associated disease, characterized by the deposition of polymeric IgA1 in the glomeruli notably on mesangial areas. It has been shown that mice expressing both human IgA1 and CD89 display features of IgAN with mesangial deposits of IgA1 and soluble CD89 (sCD89) complexes resulting in kidney inflammation, hematuria, and proteinuria [192]. Mice expressing IgA1 alone have mostly endothelial IgA1 deposits and CD89 is required to generate mesangial injury. Interestingly, transglutaminase‐2 plays an essential role in mesangial IgA‐sCD89 deposition in IgAN, possibly through its ability to cross‐link the mesangial IgA1 receptor, the transferrin receptor (CD71) [192, 193]. Soluble CD89, which is found either complexed with IgA or free, in IgAN patients can bind to CD71 directly, independently of IgA [194]. Recently, soluble CD89 was found to directly induce cell proliferation, notably of mesangial cells, which is mediated in part through binding to CD71 wit activation of the mTOR pathway [194]. Deglycosylation of IgA1, a hallmark of IgAN, has been shown to favor its binding to IgA receptors either inducing shedding of CD89 [195] or inducing overexpression of CD71 in mesangial cells [196] or enterocytes in celiac disease [172]. Together, these findings indicate that interactions between multiple actors are required for IgA‐disease development.

Increased levels of IgA‐related cytokines, such as BAFF, have been postulated in the pathogenesis of IgAN. Overexpression of BAFF in BAFF‐tg mice increased baseline levels of IgA and was associated with IgA‐dominant glomerulonephritis despite the presence of IgM deposits in the glomeruli [197]. Mouse IgA in this model was shown to be polymeric, hypoglycosylated, and reactive with intestinal commensals. Axenic conditions abrogated IgA production and protected the mice from renal disease. These data show that stimulation from an external environmental factor, namely the microbiota, is required to induce IgA‐mediated disease, even in a genetically primed animal model, and suggest a mucosal source of nephritogenic IgA [197]. However, data from IgAN patients remained inconclusive, as circulating BAFF levels have been shown to be increased in the patient's serum by some authors [198, 199, 200], but unchanged or even significantly lower compared to controls by others [197, 201, 202].

## 6 IgA and Autoimmunity

Since the identification of altered glycosylation of circulating antibodies in patients with rheumatoid arthritis in the 1980s [203], there has been increasing evidence for the role of glycans in autoimmunity. Some autoimmune diseases are strongly linked to a particular Ig class or subclass [204]. Individuals with IgA deficiency, the most common primary immune‐deficiency worldwide characterized by decreased or absent levels of serum IgA [205], have an increased incidence of allergy or autoimmune disease, particularly idiopathic thrombocytopenic purpura and arthritis. It is estimated that around 30% of IgA deficient individuals have an autoimmune disease or allergic symptoms [206], reinforcing the importance of the anti‐inflammatory role of serum IgA via FcαRI ITAMi signaling.

It is well established in antibody‐mediated autoimmunity that the antigen specificity of a given antibody will determine the site of attack, whereas the glycan/Ig isotype combination will be responsible for the physical nature of the disease [204]. Recently, IgA autoantibodies to mesangial cells targeting β2‐spectrin have been described in a subpopulation of Japanese IgAN patients [207]. Although the role of oral bacteria dysbiosis has been suspected, the cause of these auto‐antibodies remains unknown. In BAFF transgenic mice, anti‐commensal IgA antibodies were seen after colonization with pathobionts, modeling observations of a tonsil microbiota dysbiosis in patients with IgAN that showed increased carriage of the Neisseria with elevated anti‐Neisseria serum IgA in IgAN patients. The authors postulate a role for cytokine‐driven aberrant mucosal immune responses to oropharyngeal pathobionts in the immunopathogenesis of IgAN [208].

It has been hypothesized that altered glycans could cause autoimmunity, whereby each disease has a unique glycan signature characterized by the site‐specific contents of individual glycan structures present on immune cells and proteins, especially the site‐specific glycosylation patterns of the different Ig classes and subclasses [209].

**FIGURE 4:**
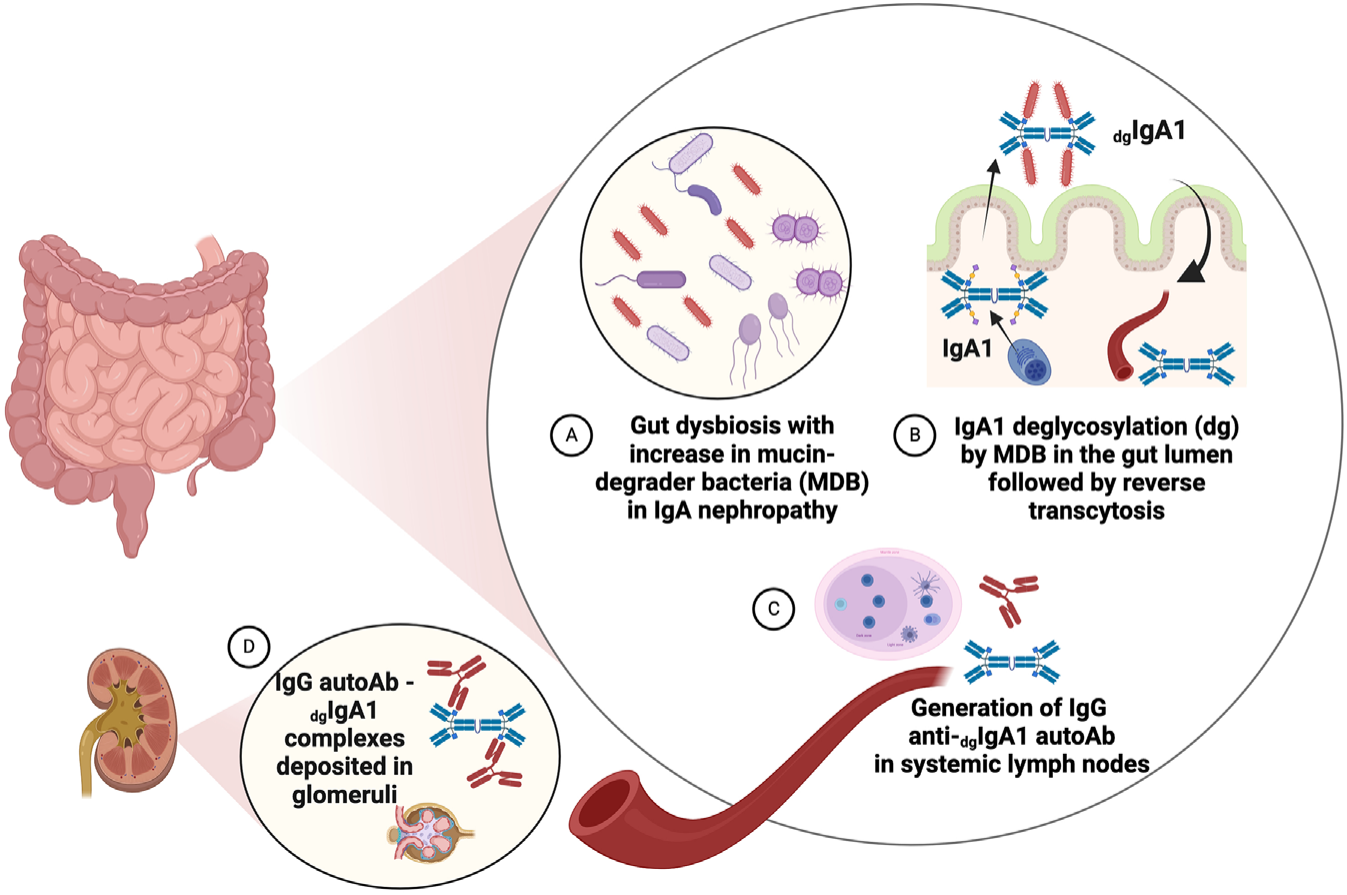
Harmful role of bacteria‐induced posttranslational deglycosylation of IgA1 in autoimmunity. (A) Mucin‐degrading bacteria have enzymes capable of digesting O‐glycans found in the mucus secreted by the intestinal epithelium, which shares motifs with the O‐glycans found on the hinge region of IgA1. Degradation of these O‐glycans makes the hinge region of IgA1 susceptible to proteolytic activity. Patients with IgAN have been shown to have increased quantities of mucin‐degrading bacteria in their gut. (B) After secretion into the intestinal lumen, the O‐glycans on the hinge‐region of IgA1 have been shown to be deglycosylated by mucin‐degrading bacteria, through the removal of sialic acid and galactose residues. This favors the reverse passage of IgA1 back across the intestinal epithelium to the circulation. (C) This modified form of IgA1 with a deglycosylated hinge region (dg‐IgA1) becomes unrecognizable as self, and a target of the systemic immune system, leading to the generation of anti‐dg‐IgA1 autoantibodies. (D) These autoantibodies then form immune complexes with dg‐IgA1 which deposit in the filtration units of the kidney (glomeruli) causing inflammation and kidney disease (glomerulonephritis). Created with BioRender.com.

Despite several descriptions of a link between Ig glycosylation and autoimmunity, no causal relationship between a given environmental factor and the appearance of an autoantigen had been established, until that recently described for IgAN [210]. IgAN pathogenesis involves aberrantly glycosylated IgA1 with galactose‐ and sialic acid‐deficient hinge regions (dg‐IgA1), that form complexes with IgG autoantibodies [58, 211], and a soluble form of the IgA Fc receptor (sCD89) [194], which deposit in the renal mesangium. Renal dg‐IgA1 deposits lead to inflammation and may also affect mesangial cell metabolism [212, 213]. Recently, it was described that a dysbiosis of commensal mucin‐degraders such as *Akkermansia muciniphila* is increased in the gut of IgAN patients [202]. This bacteria was also found in the mouse intestine of α1KICD89Tg mice (a humanized model of IgAN). Experimental approaches using this mouse model allowed proof‐of‐concept of a causal relationship between such dysbiosis and autoimmunity. In fact, gut microbiota depletion by antibiotics was able to reverse the disease in the α1KICD89Tg mice abolishing IgA1 renal deposits but without modifying total serum IgA1 levels [214]. Gut colonization with Akkermansia of microbiota‐depleted mice resulted in an aggravated phenotype of IgAN. It was shown mechanistically that IgA1 dimers were deglycosylated in the gut lumen resulting in reverse transcytosis by enterocytes of dg‐IgA1 back into the circulation by a similar mechanism as that described in Celiac disease [172]. During this process, gut lumen‐derived dg‐IgA1 in the systemic circulation becomes an autoantigen, targeted by IgG anti‐dg‐IgA1 to form nephrotoxic immune complexes that become trapped in the renal mesangium (Figure 4). Previous studies using fecal microbiota transplantation (FMT) of patient stools into antibiotic‐treated α1KICD89Tg mice demonstrated that IgAN‐associated microbiota dysbiosis induced renal IgA1 deposits and increased in dg‐IgA1 serum levels [200].

## 7 IgA as a Therapeutic Molecule

Therapeutic approaches with IgA have been developed by targeting either IgA antibody or Fcα effector functions.

### 7.1 IgA Antibody Approaches

Oral vaccines developed by Jonas Salk in the early 1950s using inactivated poliovirus vaccine (IPV) turned out to be a worldwide success in protection against poliomyelitis [215]. As IgA is the most prevalent antibody class in mucosal surfaces, enteric neutralizing IgA was found, as expected, to be the main class of antibodies against poliovirus—a major weapon for the global initiative to eradicate polio [216]. Since then, multiple approaches have been used triggering pathogen‐specific mucosal IgA responses through immunization protocols to combat viruses and bacteria [217]. For example, based on demonstrations that monoclonal IgA antibodies could protect against tuberculosis [218], vaccines using a tuberculosis protein subunit were developed. It turns out that mucosal immunization followed by intramuscular priming could induce high levels of antigen‐specific lung mucosal and systemic IgA antibodies [219]. Moreover, nasal immunization with pneumococcal surface protein A in mice was able to induce SIgA antibodies at mucosal sites and IgG antibodies systemically, both with specific anti‐pneumococcal protein A protecting these animals against colonization of *S. pneumoniae* . Mucosal induction of IgA responses has become a new challenge for fighting against pathogen immune evasion [220].

Historically, enterally administered preparations of polyclonal IgG and IgA antibodies purified from human serum have been used for the treatment of *C. difficile* colitis and, in infants, necrotizing enterocolitis [221]. Okai et al. have shown that enteral administration of an IgA monoclonal antibody modulates the intestinal microbiota in mice—bacteria recognized by the mAb were depleted [222].

The proinflammatory functions of IgA‐FcαRI‐interaction have been exploited to elicit anti‐tumor immunity. IgA‐bispecific antibodies have been proposed as novel drugs to treat cancer by enhancing activation of FcαRI‐expressing immune cells [223, 224, 225]. Of note, neutrophil FcαRI targeting by IgA was strongly effective for tumor cell killing and higher than IgG antibodies targeting IgG FcR on neutrophils. This can be explained by the fact that most of the FcRγ chain of resting neutrophils are associated almost exclusively with FcαRI [226].

IgA mAbs anti‐tumor antigens (i.e., CD20, HER2, or EGFR) are able to engage FcαRI‐expressing cells promoting cell migration to tumor area and cell cytotoxicity [227, 228]. However, technical restrictions in the production of these human IgA mAbs exits leading to altered IgA glycosylation which causes immunogenicity in the host, blocking the antibody effect. Interestingly, bispecific antibodies targeting both tumor antigens and CD89 receptors induce neutrophil migration and tumor cell killing [229]. Recent data shown that constructions generating IgG1‐IgA2 crossed antibodies, such as anti‐epidermal growth factor receptor‐2 antibodies, recruit neutrophils to the tumor site, enhancing ADCC of human breast cancer cells by neutrophils [230], representing a promising therapeutic opportunity for cancer patients.

### 7.2 Fcα Effector Approaches

IgA plays a double‐role by maintaining immune homeostasis in systemic and mucosal compartments depending on its molecular nature. While serum mIgA induces anti‐inflammatory signals through its Fc fragment bound to FcαRI, followed by recruitment of phosphatases to dampen excessive immune responses, IgA‐IC in IgA‐associated autoimmune diseases and infection with IgA‐associated pathogens result in proinflammatory responses. The manipulation of the IgA Fc interaction with FcαRI offers several therapeutic strategies for the treatment of inflammatory diseases.

Inducing ITAMi inhibitory signaling by targeting FcαRI with mIgA on myeloid cells has been demonstrated to prevent disease development in several inflammatory disease mouse models [8]. Initial studies were performed using Fab fragments of mouse monoclonal antibodies directed to the EC2 domain of FcαRI. Anti‐FcαRI Fab fragments induced ITAMi signaling due to their monovalent targeting which in mouse models could prevent the development of asthma, glomerulonephritis, and sterile inflammation induced by the ureteral obstruction model [126, 130]. These findings demonstrated that anti‐FcαRI Fab could be used as a new therapeutic tool to prevent the progression of inflammatory diseases. To determine whether mIgA treatment could also be beneficial to prevent or reverse an established inflammatory disease, studies using highly purified mIgA obtained from thousands of blood donors during IVIg production provided by an industrial partner (CSL Behring, Bern, Switzerland) were performed [137]. Treatment with this mIgA impaired in vitro cell activation in a CD89‐FcRγ‐dependent manner through ITAMi signaling, recruiting tyrosine phosphatase SHP‐1. Human mIgA was highly effective in either preventing or reversing established experimental arthritis (CAIA or CIA model developed in CD89‐transgenic mice). mIgA markedly blocked the recruitment of tissue leukocyte recruitment to inflamed joints of mice, linked to tissue induction of SHP‐1 phosphorylation. Importantly, ex vivo studies of the synovial fluid from rheumatoid arthritis patients allowed the formal demonstration of human mIgA inhibitory action by reversing cellular inflammation through the induction of an ITAMi configuration. Another approach is presently being conducted by the biotech company Inatherys using humanized monovalent Fab or divalent F(ab')_2_ fragments of anti‐CD89 mAbs which induces ITAMi signaling, allowing an alternative approach to treat inflammatory diseases as previously demonstrated in mouse models [8]. Together, these results indicate the promising therapeutic potential of anti‐CD89 mAbs that mimic mIgA or highly purified human mIgA [231] as new anti‐inflammatory biologics.

Finally, IgG mAbs that block IgA‐FcαRI interactions have recently been generated for therapeutic use [232], for which the results from ongoing studies seem promising (JJP biologics). These anti‐CD89 mAbs bind to different epitopes on EC1 of CD89 where IgA‐binding sites are located, and have the capacity to block IgA‐mediated functions.

## 8 Concluding Remarks

IgA works at the coal face of host–environment immune interactions. The magnitude of resources invested by the body in the daily production of IgA attests to its essential role in immune homeostasis. Its molecular form is partitioned according to function in different immune compartments, while its overarching purpose is to mitigate inflammatory responses and promote tolerance. However, when circumstances require, such as with pathogen invasion, IgA can collaborate with effector mechanisms that mediate potent inflammatory responses.

The role of polymeric mucosal IgA in protection against invading pathogens has already been exploited with great effect through vaccination. The importance of mucosal IgA as a curator of symbiotic relationships with the microbiota, particularly in early life, is just beginning to be appreciated and could pave the way toward therapeutic interventions. Anti‐inflammatory effects of mIgA in the circulation hold promise for the treatment of inflammatory diseases.

Studying the glycosylation of IgA has given insight into mechanisms of autoimmunity, as seen in IgAN. These observations should stimulate further investigation of glyco‐immunological processes in other diseases.

Continuing to decrypt the workings of the IgA immune system will not only give further insight into the regulation of inflammatory responses but also help understand our interactions with the environment and how this can shape both health and disease.

## Conflicts of Interest

P.J.G. is a medical and/or scientific advisor to Calliditas Therapeutics. R.C.M. reports grants from Moderna, Shire, and Biomarin. R.C.M. and P.L. are co‐founders of Inatherys. N.O.S.C. and A.L. do not declare any conflicts of interest.

## Data Availability

Data sharing is not applicable as no new data were generated.
